# Monocyte Phenotypes and Physical Activity in Patients with Carotid Atherosclerosis

**DOI:** 10.3390/antiox11081529

**Published:** 2022-08-05

**Authors:** Mathilde Mura, Michèle Weiss-Gayet, Nellie Della-Schiava, Erica Chirico, Patrick Lermusiaux, Marie Chambion-Diaz, Camille Faes, Anaelle Boreau, Bénédicte Chazaud, Antoine Millon, Vincent Pialoux

**Affiliations:** 1 Interuniversity Laboratory of Human Movement Biology EA7424, Université Claude Bernard Lyon 1, Université de Lyon, 69008 Lyon, France; 2 Institut NeuroMyoGene, CNRS UMR 5310, INSERM U1217, Université Claude Bernard Lyon 1, Université de Lyon, 69008 Lyon, France; 3 Department of Vascular and Endovascular Surgery, Louis Pradel Hospital, 69008 Lyon, France; 4 Department of Biomedical Sciences, Cooper Medical School of Rowan University, Camden, NJ 08103, USA; 5 Institut Universitaire de France, 75000 Paris, France

**Keywords:** atherosclerosis, inflammation, physical activity, monocytes, cytometry, GPAQ

## Abstract

Atherosclerosis is associated with low-grade inflammation involving circulating monocytes. It has been shown that the levels of intermediate pro-inflammatory monocytes are associated with cardiovascular mortality and risk of ischemic stroke. It also has been shown that physical activity (PA) decreases inflammation markers, incidence of strokes, and mortality. In this cross-sectional study, we tested the effect of PA on circulating monocytes phenotype rate. A total of 29 patients with a carotid stenosis > 50% were recruited. Levels of physical activity (MET.min/week) were measured by the GPAQ questionnaire, arterial samples of blood were collected to analyze monocyte phenotype (classical, intermediate and non-classical) assessed by flow cytometry, and venous blood samples were used to dose antioxidant activity and oxidative damage. Antioxidant capacity was reduced and oxidative damage increased in patients. There was a significant decrease in the percentage of classical and intermediate monocytes in moderately active patients as compared with non-active and highly active patients. Inversely, the rate of non-classical monocytes increased in moderately active patients. Intense PA appears to blunt the beneficial effects of moderate PA. Our study also suggests that PA could be beneficial in such patients by reducing the rate of intermediate monocytes known to predict the risk of ischemic stroke and by increasing the non-classical monocytes involved in lesions’ healing. Nevertheless, a longitudinal study would be necessary to confirm this hypothesis.

## 1. Introduction

Chronic physical activity (PA) is likely one of the most powerful non-surgical therapies against the progression of cardiovascular diseases. More specifically, PA has been shown to be independently associated with lower odds of carotid artery stenosis and peripheral artery disease [1]. Currently, the rupture of vulnerable carotid plaques remains one of the main causes of ischemic stroke worldwide, which may lead to physical impairment or death of the patient. Inflammation and oxidative stress are two major processes involved in atherogenesis and plaque vulnerability [2]: indeed, vulnerable plaques contain immune cells trying to phagocytize oxidized low-density lipoproteins (oxLDL) [3]. Moreover, atherosclerotic patients present with chronic low-grade inflammation often evolving over the course of decades [4]. Circulating monocytes are closely related to plaque formation and destabilization [5], although monocyte subsets appear to be unequally involved [6,7,8,9] in plaque pathophysiology. Classical monocytes (cluster of differentiation: CD14^++^/CD16^−^) and intermediate monocytes (CD14^++^/CD16^+^) initiate inflammatory atherosclerotic lesions leading to plaque vulnerability [7] and are associated with increased ischemic risk. On the contrary, non-classical monocytes (CD14^+^/CD16^++^) limit the atherogenesis process and may play a role in the resolution of inflammation within the plaque. Indeed, a study suggested that circulating monocyte phenotype may influence lesional macrophage polarization in heathy subjects and in patients with vascular diseases [10,11]. Differences in macrophage phenotypes within the plaque modulate plaque vulnerability [12,13]; it also appears that this circulating monocyte phenotype could affect plaque features and be involved in plaque vulnerability [6,9,13]. Chronic PA was shown to modulate circulating monocyte phenotype in healthy subjects [14,15,16], and it has been suggested that such a mechanism could be involved in the protective effect of PA seen in atherosclerosis patients [7]. Previous work implied that a reduction of inflammation due to PA [17] might be mediated by circulating monocyte count and differences in phenotype in old people [18]. Moreover, our team previously reported that PA was associated with a reduced risk of intraplaque hemorrhage in carotid atherosclerotic patients [19]. Despite large advances in the understanding of the pathophysiology and management of vulnerable carotid plaques, the effects of PA (i.e., intensity, duration, and frequency) on monocyte phenotype in patients with atherosclerotic plaques are unknown and need to be investigated [7]. Therefore, understanding the effect PA and its impact on monocyte subsets is necessary to optimize future modalities of PA as therapy in such patients. 

## 2. Methods

### 2.1. Ethics and Population

The present study has been approved by the National Ethics Committee (ID RCB: 2019-A01543-54/SI: 19.06.21.40640), the «Agence Nationale de Sécurité du Médicament et des produits de santé» and registered at the “Commission Nationale de l’informatique & Libertés” 19-366. This study is registered on http://www.clinicaltrials.gov (NCT number: NCT04053166), accessed on 1 October 2019. All patients gave informed consent. A total of 29 patients presenting a carotid stenosis ≥50% and 14 healthy subjects were included. Patients were white, asymptomatic, or symptomatic from stroke and transient ischemic attack. They did not have cancer or heart failure, nor were they seropositive. They all had individually optimized drug treatments.

### 2.2. Data Collection

#### 2.2.1. Questionnaires

Patients completed the following questionnaires. Cognitive capacities were evaluated using the Folstein mini-mental questionnaire [20,21]; diet was characterized with the National Nutrition Health Plan questionnaire [22,23]; smoking habits were collected in order to establish a pack-year consumption rate; sedentary behavior was evaluated using the sedentary behavior Questionnaire [24], and PA duration (min/week), intensity (MET), and levels (MET.min/week) were determined with the Global Physical Activity Questionnaire [25,26]. An in-house nutrition questionnaire was used to asses food consumption, rating food intake frequency and quality (processed food, fruits, vegetables, meat, sweets, and alcohol) on a scale from 1 to 3, with 1 being the best habit and 3 the worst. Healthy subjects filled in the same questionnaires, with the exception of the Folstein mini-mental questionnaire. 

#### 2.2.2. Monocytes Analysis

**Figure 1:**
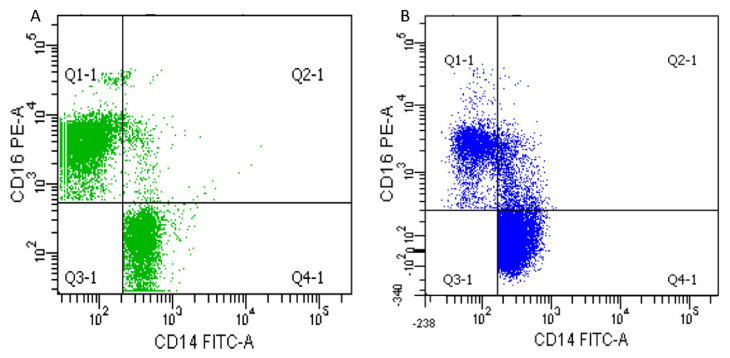
Representative scatter plots for CD14:FITC and CD16:PE were used to determine the percentage of each phenotype by flux cytometry. Q4-1 contains the classical monocytes (CD14^++^CD16^−^), Q2-1 contains the intermediate monocytes (CD14^++^CD16^+^), and QI-1 contains the non-classical monocytes (CD14^+^CD16^++^). (**A**) Typical scatter plot of a patient from the group 2 (1600 MET.min/sem–4500 MET.min/sem); (**B**) Typical scatter plot from a patient from the group 3 (>4500 MET.min/sem).

After an overnight fast, venous blood was taken at rest in heparin tubes for healthy subjects and patients. Peripheral blood mononuclear cells (PBMCs) were isolated from the whole blood over a Ficoll (Ficoll Paque PREMIUM, Cytiva, UK) gradient. The labelling procedure of PBMC included incubation with Fc receptor blocking solution (FcR Blocking Reagent human, Miltenyi biotech, Germany) for 30 min to saturate monocyte Fc receptors. Then, FITC-conjugated anti-CD14, PE-conjugated anti-CD16 (both Miltenyi biotech, Germany) antibodies were added for 30 min at 4 °C. Controls included isotype-matched antibodies. A total of 50,000 monocytes were discriminated from all events screened into the flow cytometer based on size, granularity and cell surface markers and then analyzed using a BD FACSCanto II (Beckton Dickinson, NJ, USA) flow cytometer (Figure 1). 

#### 2.2.3. Oxidative Stress and Antioxidants Assays

After an overnight fast, venous blood was taken at rest in EDTA tubes and were immediately centrifuged and stored at −80 °C until analysis. Blood plasma samples were assessed for markers of oxidative stress (advanced oxidation protein products [AOPP] and malondialdehyde, [MDA]) and antioxidant enzymes activity (catalase; glutathione peroxidase [GPx] and superoxide dismutase [SOD]). Plasma superoxide dismutase (SOD) activity was at 450 nm by measuring the degree of inhibition of the reaction between superoxide radical (O_2_^•−^) and nitroblue tetrazolium; a colorimetric molecule that competes with SOD for O_2_^•−^ [27]. Catalase catalyzes the reaction between hydrogen peroxide (H_2_O_2_) and methanol, producing formaldehyde, which can be quantified at 540 nm using a chromogen. Activity was compared to a standard of formaldehyde apparition [28]. Glutathione peroxidase (GPx) activity was indirectly determined over a 5 min period at 340 nm by the rate of oxidation of NADPH to NADP^+^ [29]. Malondialdehyde (MDA) concentration, marker of the lipids oxidation, was quantified using 1,1,3,3-tetraethoxypropane as a standard by measuring the apparition of a pink chromogen representative of the formation of a MDA-thiobarbituric acid product [30] using photometric absorption at 532 nm. Advanced oxidation protein product (AOPP) levels were quantified against a standard of chloramine-T, potassium iodide and acetic acid at 340nm and expressed as μmol/L of chloramine-T equivalents [31]. 

#### 2.2.4. Clinical Data

Personal medical history of the patients (i.e., ischemic event record, body mass index, type 2 diabetes, dyslipidemia, hypertension) was documented. Complete blood count was reported in order to assess total leucocytes, monocytes, neutrophils, and lymphocytes count, fibrinogen levels, and lipid profile (i.e., total cholesterol, HDL cholesterol, LDL-cholesterol and triglycerides).

### 2.3. Statistics

Quantitative variables were expressed as mean ± standard deviation, and categorical data were presented as occurrence and frequency in each group. PA was evaluated in duration (minutes/week), intensity (4 vs. 8 Metabolic equivalents of Task: METs) and level (MET.min/week), and were then classified in three groups according to PA threshold on the cardiovascular mortality and morbidity: less than 1600 MET.min/week (group 1) between 1600 and 4500 MET.min/week (group 2), or exceeding 4500 MET.min/week (group 3). Above 1600 MET.min/week, overall, cardiovascular-, stroke and ischemic heart disease-related deaths have been shown to be impacted [32]. Above the threshold of 4500 MET.min/week no more benefits from moderate to vigorous PA is observed on mortality rates [33]. Comparisons of monocyte phenotype variations between PA groups were done by parametric tests (ANOVA) if the data were normally distributed, and the homogeneity of variances were respected. If not, non-parametric tests (Kruskal–Wallis or Wilcoxon test, as appropriate) were performed. The significance level was set at *p* < 0.05. Post-hoc comparisons were corrected for multiple comparisons. All statistical analyses were performed using Rstudio software (version 1.8.2, Boston, MA, USA). 

## 3. Results

### 3.1. Populations Characteristics

Patients were 71 ± 9 years-old, the gender ratio was 22/7 men/women, and the mean body mass index (BMI) was 25 ± 3 kg/m^2^. The healthy control group was 36 ± 9 years-old (difference with patient group: *p* < 0.0001), they were all male, and the mean BMI was 24 ± 3 kg/m^2^ (ns). In the patient group, the mean carotid plaque stenosis was 70.9 ± 9.8%, and 14 patients were symptomatic of an ischemic event. Seven patients had diabetes mellitus, 9 presented with dyslipidemia, and 19 had hypertension. Overall, 20 patients were treated with statins, and 21 took anti-platelets drugs. Healthy subjects were free of all the preceding comorbidities and drug treatments. The tobacco consumption of the patients was 31 ± 30 pack-years, whereas for healthy subjects was 1 ± 2 pack year (*p* < 0.001). There was no difference between the control group and the patients for sedentary behavior (respectively 556 ± 161 and 530 ± 177 min/day) and PA duration (respectively 3204 ± 1632 and 2775 ± 2064 MET.min/week). The nutrition score was lower for patients (1.4 ± 0.3 UA) in comparison with healthy subjects 1.6 ± 0.3 UA (*p* < 0.05).

### 3.2. Comparison between PA Groups and with the Healthy Subject Group

The monocyte subset distribution was 46.4 ± 16.9% for classical monocytes, 4.8 ± 2.6% for intermediate monocytes, and 50.5 ± 15.7% for non-classical monocytes in the patient population; no significant difference was seen with the healthy control group (38.7 ± 8; 6.0 ± 1.8, and 55.3 ± 8.4%, respectively, for each monocyte subset). The catalase enzyme activity was significantly lower in patients than in healthy subjects (−48%, *p* < 0.0001). MDA concentration was higher in the patient group in comparison with the healthy subject group (+33%, *p* < 0.001), as well as AOPP levels (+82%, *p* < 0.0001; Table 1).

There were no differences in complete blood count, risk factors, and comorbidities across PA level groups; however, there were significant differences noted for PA intensity (Table 2) (i.e., prevalence of patients practicing at 8 METS was 0 %, 22 %, and 71 % for group 1, 2, and 3, respectively). 

**Figure 2:**
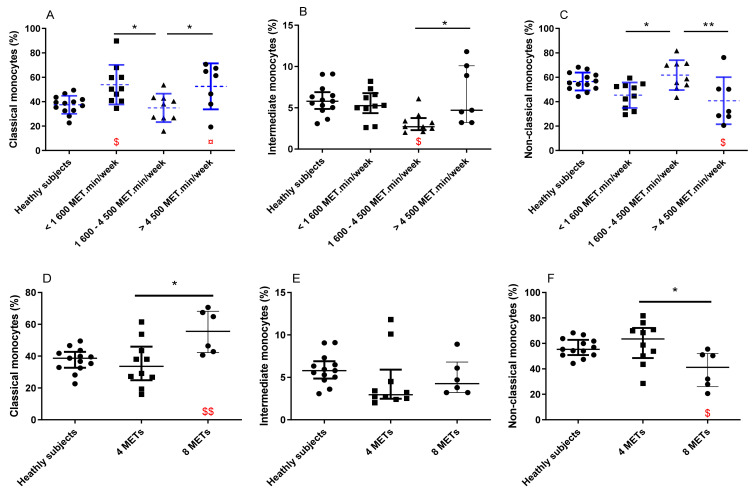
Monocyte phenotypes for each PA level groups (**A**–**C**) and PA intensity group (**D**–**F**), in comparison with healthy subjects. Patients were classified in three groups depending on the weekly level of PA (group 1: *n* = 10; 586 ± 377 MET.min/week; group 2: *n* = 9; 3233 ± 781 MET.min/week; and group 3: *n* = 7; 5457 ± 757 MET.min/week). (**A**) Classical monocytes frequency; (**B**) intermediate monocytes frequency; and (**C**) non-classical monocytes frequency. Patients were stratified in two groups depending on the weekly intensity of PA (moderate group: *n* = 9, involved in moderate (4 MET.min/week) PA; intense group: *n* = 6 involved in moderate to intense (4 MET.min/week to 8 MET.min/week) PA. (**D**) Classical monocytes frequency; (**E**) intermediate monocytes frequency; and (**F**) non-classical monocytes frequency. Rate of each monocytes phenotype is expressed as a percentage of the total monocytes count. Parametric statistical analysis was done on data represented on graph (**A**,**C**), thus, mean and standard deviation are represented in dashed and blue lines. Non-parametric statistical analysis was done on data represented on graph (**B**,**D**–**F**), thus, median and interquartile range are represented in continuous black lines. MET: Metabolic Equivalent of Task; min: minute; PA: Physical activity. * *p* < 0.05 between patient groups. ** *p* < 0.01 between patient groups. In red: ¤ *p* = 0.06 difference with the healthy donor group. $ *p* < 0.05 difference with the healthy donor group. $$ *p* < 0.01 difference with the healthy donor group.

There was a decrease in the percentage of classical monocytes (*p* = 0.02 and *p* = 0.03, respectively) and intermediate monocytes (*p* = 0.13 and *p* = 0.05, respectively) in group 2 as compared with groups 1 and 3 (Figure 2A,B). The non-classical monocytes rate was increased in PA level group 2 as compared to groups 1 and 3 (*p* = 0.02 and *p* = 0.008, respectively; Figure 2C). Classical monocyte percentage was increased in groups 1 and 3 in comparison with the healthy subject group. In comparison with healthy subjects, intermediate monocyte rate was reduced in group 2, and non-classical monocytes percentage was reduced in group 3. Regarding the antioxidant enzymes (SOD, catalase, and GPx) activity, no difference was observed between the patients’ PA level groups. Yet, all the groups of patients had a reduced activity of catalase (−46%, *p* < 0.01; −37%, *p* < 0.05 and −62%, *p* < 0.001 for groups 1, 2, and 3, respectively) in comparison with the control group. No significant differences were observed between the groups for MDA and AOPP. Nevertheless, only active patients (group 2 and group 3) had an increased level of MDA (+32%, *p* < 0.05 and +39%, *p* < 0.01 for groups 2 and 3, respectively) in comparison with healthy subjects. Patients from PA groups 1 and 2 had increased levels of AOPP (+103%, *p* < 0.001 and +101%, *p* < 0.001 for group 1 and 2, respectively) in comparison with healthy subjects (Table 1).

To explain this unexpected U-shaped relationship between monocyte subset distribution and PA level, we hypothesized that the beneficial effect of PA may be blunted with high intensity exercise. We thus conducted a separate analysis on the most active patients (groups 2 and 3). These patients were divided into two groups according their PA intensity: moderate (MPA group: 4 METs) vs. intense (IPA group: 8 METs) PA practice groups, which were then compared. The two PA groups were equivalent in complete blood count, risk factors, and comorbidities, by design, PA intensity was the only variable that was modified between groups (Table 3). Patients practicing intense PA had an increased number of classical monocytes and intermediate monocytes in blood samples (Figure 2D), associated with a decreased number of non-classical monocyte production in comparison with patients practicing moderate PA (Figure 2F). In comparison with healthy subjects, patients practicing intense PA had increased rates of classical monocytes and decreased rates of non-classical monocytes (Figure 2D,F). No difference was observed between the two PA groups for the antioxidant enzymes (SOD, catalase and GPx). Yet, both intensity groups of patients had a reduced activity of catalase (−50%, *p* < 0.001 and −47%, *p* < 0.01 for MPA and IPA group, respectively) in comparison with the control group. Patients practicing moderate and intense PA had increased levels of MDA (+26%, *p* < 0.05 and +52%, *p* < 0.01 for MPA and IPA group, respectively) in comparison with the control group. Patients practicing moderate PA had an increased concentration of AOPP (+80%, *p* < 0.01) in comparison with the control group (Table 1).

## 4. Discussion

As it was previously suggested, the monocyte phenotype in patients with atherosclerotic plaques needed to be investigated [7]. Our study reports for the first time that moderate intensity PA decreases the number of classical and intermediate monocytes in circulation of carotid atherosclerotic patients, whereas intense PA reverses the beneficial effects of moderate PA. These results bring new information regarding the guidelines of PA practices in this elderly atherosclerotic population.

In Europe, life expectancy at birth has increased to 70 years old, resulting in an increase in the elderly population [34]. According to a recent meta-analysis, at least half of males aged 65 years old and older have carotid plaques [35] and surgery appears to be unnecessary in the majority of cases [36]. This emerging population is currently understudied, but as it is growing, it needs to be better understood in order to improve lifestyle management guidelines. The link between systemic low-grade inflammation and carotid atherosclerotic lesions is well established [4,6,9,37], however, little is known about differential monocyte phenotype effects in human atherosclerotic lesions [38].

Increased oxidative stress is a well-known mechanism that leads to atherogenesis [39]. Our results show that patients have decreased antioxidant catalase activity, thus, they are less protected against the increase in oxidative stress inherent to aging [40]. Therefore, our patients showed increased lipids (MDA) and protein (AOPP) oxidative damage in comparison with healthy subjects. Our results also suggest that patients have a monocyte phenotype distribution that does not differ from healthy subjects. High rates of classic and intermediate monocyte subsets were associated with an elevated risk of cardiovascular events in this specific population with atherosclerosis [6,9,13], like our groups of patients with carotid plaque over 50% carotid stenosis. However, the monocytes phenotype rate in healthy people is, therefore, likely not relevant to the risk of cardiovascular event when not associated with atherosclerosis or other cardiovascular risk factors. The lack of PA level effect on monocytes phenotype rate in healthy patients (results not shown) strengthens this hypothesis.

To our knowledge, the influence of PA on circulating monocyte phenotype has never been analyzed in carotid atherosclerotic patients. Several studies tried to characterize the monocyte phenotype in response to exercise, but these studies were done before the 2010s and thus, did not make the distinction between intermediate and non-classical monocytes [14,41]. The decrease in classical and intermediate monocytes for the 1600–4500 METs.min/week group may result from a systemic down-regulation of pro-inflammatory pathways [42,43]. Indeed, long-term moderate exercise downregulates the PBMC production of atherogenic cytokines and upregulates the production of the atheroprotective ones in subjects at risk of ischemic disease [44]. Alterations in the lipid metabolism resulting in increased oxLDL is an important component of atherogenesis and plaque vulnerability [45], thus, the decrease in circulating oxLDL in response to PA previously observed in populations at risk of vascular disease [46] may blunt the pro-inflammatory shift of monocytes [7]. 

Although the response to moderate PA in our population is consistent with contemporary work with sedentary elderly people [16], the response to intense PA could be viewed as counterintuitive since high intermittent intensity training shows greater efficacy on cardiorespiratory fitness as compared to moderate intensity training in patients with cardiovascular diseases [47]. Indeed, in our patient population, in comparison with healthy subjects, low PA levels and moderate PA practice appears to induce more oxidative damage on proteins (i.e., AOPP) than high PA practice and high PA levels, and intense PA practice appears to enhance more oxidative damage on lipids (i.e., MDA) compared to low PA level. Yet, it is known that an increased concentration of oxLDL promotes a pro atherogenic shift in classical monocytes [48]. Thus, the increase in classical monocytes in the IPA group could be related to its greater increase in lipid damage. These activated monocytes are known to produce superoxide anion through NADPH oxidase, increasing the pro-oxidant state in circulation and in atherosclerotic plaque [49]. In this perspective, it would be interesting for future studies to determine the difference in NADPH oxidase expression in activated monocytes between healthy and atherosclerotic patients and in the function of PA level. In addition, oxidized lipids’ incubation with monocytes was shown to increase their release in reactive oxygen species and proinflammatory cytokines [50]. However, the very modest difference in our oxidative stress markers between PA groups of patients could also suggest that the monocytes phenotype rate modulation by physical activity level in patients with atherosclerosis could be independent of oxidative stress and more related to the changes of other biological pathways, as inflammation. Systemic pro-inflammatory effects are well described in a transitory period after the practice of intense PA [51]. Usually, in healthy subjects, this pro-inflammatory reaction is quickly reversed [52]. Thus, the increase in classical and intermediate monocytes that we observed in patients practicing intense PA (vs. moderate intensity PA) may result in an exaggerated or prolonged pro-inflammatory response in patients with atherosclerosis. Low-grade inflammation is a well-known circulatory process associated to atherosclerosis [4,53,54], sustained by cytokines secreted by plaque and circulating leukocytes [55]. Thus, the secretion of additional pro-inflammatory cytokines induced by the practice of PA [56] may result in a cytokine overload, thus exacerbating the circulating pro-inflammatory environment [55] in alignment with the increased rates of classical monocytes that were observed. Our results suggest that intense PA compared to moderate PA may deteriorate monocyte profiles and should be considered with relation to another study reporting no beneficial effect of PA on cardiovascular mortality when practicing more than 4500 METs.min/week, in contrast to lower PA level [33]. These present results should also be analyzed within the context of the results of our previous study with the same patients with carotid stenosis over 50%, which reported that patients practicing moderate PA (corresponding to our group 2) reduce the intra-plaque hemorrhage comparing to patients practicing very low PA (corresponding to our group 1) [19]. Taken together, all these results are in favor of moderate PA practice for this population. 

Although these results bring new knowledge on an understudied population, it should be acknowledged that our study presents a few limitations. First, our results are based on a cross-sectional study design. In the future, longitudinal studies assessing PA interventions should be done in order to specify the effect of PA on immune inflammatory in carotid atherosclerotic patients. Moreover, this study did not allow for determining the biological pathways associated with monocytes phenotype modulation by PA. The assessment of circulating OxLDL and cytokines in addition to more specific monocytes pro-oxidant markers may help to answer this question. Secondly, the significant difference in age between patients and healthy subjects does not allow to discriminate the sole effect of the disease from aging, thus, adding an age-matched control group might be interesting to do in future studies. Indeed, we did not match the control group in age since a majority of elderly people have undiagnosed carotid plaque [35], implying that a perfect control group without carotid plaque of a similar age (over 60 years old) is impossible to find. Patients were older, thus they smoked for longer than healthy subjects. Nevertheless, it should be underlined that control and patient groups were equivalent in time spend sedentary, total PA practice, and BMI. Finally, there were no women in the control group, whereas there were 24% in the patient group. However, all female patients were post-menopausal, thus, in order to lift hormonal interaction, only men were included in the control group. 

## 5. Conclusions

In conclusion, in atherosclerotic patients, moderate PA likely decreases pro-inflammatory monocytes, which are known to be related to plaque vulnerability, suggesting that this type of PA may be encouraged in this population. 

## Figures and Tables

**Table 1:** Antioxidant enzymes activity and oxidative stress damage in PA level groups (group 1, 2, and 3) and intensity groups (MPA and IPA) in comparison with healthy subjects. Parametric statistical tests are expressed as mean and standard deviation (written in blue), and non-parametric statistical tests are expressed as median and interquartile range (written in black). AOPP: advanced oxidation protein products; CAT: catalase; GPX: glutathione peroxidase; IPA: intense physical activity; MDA: malondialdehyde; MPA: moderate physical activity; SOD: superoxide dismutase. *** *p* < 0.001 between all patients and control group. **** *p* < 0.0001 between all patients and control group. µ *p* < 0.05 between PA level group and control group. µµ *p* < 0.01 between PA level group and control group. µµµ *p* < 0.001 between PA level group and control group. ¤ *p* < 0.05 between PA intensity group and control group. ¤¤ *p* < 0.01 between PA intensity group and control group. ¤¤¤ *p* < 0.001 between PA intensity group and control group.

	Healthy Control Group (*n* = 14)	All Patients (*n* = 29)	Group 1 (*n* = 10)	Group 2 (*n* = 9)	Group 3 (*n* = 7)	MPA (*n* = 10)	IPA (*n* = 6)
Antioxidant enzymes activity							
SOD (µmol/mL/min)	34.6 ± 6.2	32.8 ± 6.9	31.0 ± 5.4	33.3 ± 7.3	34.2 ± 8.4		
	36.9 ± 10.7					36.1 ± 13.1	31.1 ± 9.8
CAT (µmol/mL/min)	1.7 ± 0.5	0.9 ± 0.4 ****	0.9 ± 0.5 µµ	1.1 ± 0.2 µ	0.7 ± 0.3 µµµ		
	1.8 ± 0.8					0.9 ± 0.5 ¤¤¤	0.9 ± 0.9 ¤¤
GPX (µmol/mL/min)	7.6 ± 1.3	7.7 ± 1.6	7.8 ± 1.5	7.5 ± 0.6	7.3 ± 1.6		
	7.4 ± 1.7					7.7 ± 1.2	7.4 ± 2.1
Oxidative damage							
MDA (µmol/mL)	46.0 ± 8.7	59.0 ± 9.5 ***	59.2 ± 10.8	60.7 ± 9.2 µ	64.0 ± 21.2 µµ		
	47.0 ± 8.7					59.4 ± 16.6 ¤	65.5 ± 37.3 ¤¤
AOPP (µmol/mL)	91.1 ± 34.6	165.6 ± 54.9 ***	185.0 ± 55.6 µµµ	182.8 ± 32.3 µµµ	128.6 ± 56.2		
	95.7 ± 68.5					161.8 ± 117.4 ¤¤	150.6 ± 82.1

**Table 2:** Physical activity level groups risk factors, comorbidities, and complete blood count. BMI: body mass index; PA: physical activity and ns: non-significant.

	Group 1 (*n* = 10)	Group 2 (*n* = 9)	Group 3 (*n* = 7)	*p*- Value
Age (years old)	70.1 ± 6.8	71.6 ± 10.0	70.4 ± 12.6	ns
BMI	24.3 ± 3.2	26.6 ± 2.7	24.6 ± 4.3	ns
Asymptomatic/Symptomatic	5/7	5/4	5/3	ns
Stenosis (%)	73.4 ± 14.5	77.3 ± 7.6	67.9 ± 12.5	ns
Type 2 diabetes (*n*)	2	2	3	ns
Dyslipidaemia (*n*)	2	4	3	ns
Hypertension (*n*)	5	8	6	ns
Statin use (*n*)	7	8	5	ns
Anti-agregant use (*n*)	8	8	5	ns
Intense PA practice (min/week)	0 ± 0	80.0 ± 158.8	120.8 ± 149.0	*p* < 0.01
Sedentary behaviour (min/week)	525.7 ± 144.0	494.2 ± 161.8	485.4 ± 129.0	ns
Smoking habit (pack-year)	20.7 ± 30.7	33.1 ± 34.6	29.7 ± 24.0	ns
Nutrition score (UA)	1.5 ± 0.4	1.3 ± 0.2	1.3 ± 0.3	ns
Leucocytes (×10^9^)	8.9 ± 3.5	7.7 ± 2.1	7.5 ± 2.4	ns
Monocytes (×10^9^)	0.7 ± 0.2	0.6 ± 0.2	0.8 ± 0.3	ns
Neutrophils (×10^9^)	5.6 ± 2.7	5.1 ± 1.6	4.6 ± 1.6	ns
Lymphocytes (×10^9^)	2.2 ± 0.8	1.7 ± 0.6	1.9 ± 0.8	ns
Fibrinogen (g/L)	3.6 ± 0.9	4.0 ± 0.9	3.7 ± 0.8	ns
Total cholestérol (mmol/L)	5.7 ± 1.5	4.4 ± 1.7	4.0 ± 1.1	ns
HDL cholesterol (mmol/L)	1.1 ± 0.3	0.9 ± 0.3	1.1 ± 0.3	ns
LDL cholesterol (mmol/L)	3.9 ± 1.2	2.7 ± 1.2	2.3 ± 0.9	ns
Triglycerides (mmol/L)	1.5 ± 1.0	1.8 ± 0.8	1.4 ± 0.4	ns

**Table 3:** Physical activity intensity groups risk factors, comorbidities, and complete blood count. BMI: body mass index; PA: physical activity and ns: non-significant.

	Moderate PA Group (*n* = 10)	Intense PA Group (*n* = 6)	*p*-Value
Age (years old)	75.4 ± 10.6	71.5 ± 5.7	ns
BMI	26.0 ± 3.3	24.4 ± 2.9	ns
Asymptomatic/Symptomatic	6/4	3/2	ns
Stenosis (%)	75.2 ± 10.5	68.6 ± 11.2	ns
Type 2 diabetes (*n*)	3	1	ns
Dyslipidaemia (*n*)	3	2	ns
Hypertension (*n*)	7	4	ns
Statin use (*n*)	9	4	ns
Anti-platelets use (*n*)	9	3	ns
Intense PA practice (min/week)	1.8 ± 6.0	285.0 ± 113.2	*p* < 0.01
Sedentary behaviour (min/week)	485.3 ± 159.4	468.0 ± 113.9	ns
Smoking habit (pack-year)	28.6 ± 35.28	31.6 ± 22.2	ns
Nutrition score (UA)	1.3 ± 0.2	1.3 ± 0.1	ns
Leucocytes (×10^9^)	7.7 ± 1.9	7.8 ± 2.9	ns
Monocytes (×10^9^)	0.6 ± 0.2	0.8 ± 0.3	ns
Neutrophils (×10^9^)	5.0 ± 1.4	4.9 ± 2.0	ns
Lymphocytes (×10^9^)	1.9 ± 0.7	1.8 ± 0.5	ns
Fibrinogen (g/L)	1.9 ± 0.7	1.8 ± 0.5	ns
Total cholestérol (mmol/L)	4.4 ± 1.5	3.8 ± 1.0	ns
HDL cholesterol (mmol/L)	1.0 ± 0.3	0.9 ± 0.3	ns
LDL cholesterol (mmol/L)	2.6 ± 1.2	2.2 ± 0.7	ns
Triglycerides (mmol/L)	1.6 ± 0.7	1.5 ± 0.6	ns

## Data Availability

The data presented in this study are available in the article.

## References

[1] Stein R.A., Rockman C.B., Guo Y., Adelman M.A., Riles T., Hiatt W.R., Berger J.S. (2015). Association Between Physical Activity and Peripheral Artery Disease and Carotid Artery Stenosis in a Self-Referred Population of 3 Million Adults. Arterioscler. Thromb. Vasc. Biol..

[2] Mury P., Chirico E.N., Mura M., Millon A., Canet-Soulas E., Pialoux V. (2018). Oxidative Stress and Inflammation, Key Targets of Atherosclerotic Plaque Progression and Vulnerability: Potential Impact of Physical Activity. Sports Med..

[3] Chistiakov D.A., Orekhov A.N., Bobryshev Y.V. (2015). Contribution of neovascularization and intraplaque haemorrhage to atherosclerotic plaque progression and instability. Acta Physiol..

[4] Ross R. (1999). Atherosclerosis as an inflammatory disease. N. Engl. J. Med..

[5] Ley K., Miller Y.I., Hedrick C.C. (2011). Monocyte and Macrophage Dynamics During Atherogenesis. Arterioscler. Thromb. Vasc. Biol..

[6] Jaipersad A.S., Lip G.Y., Silverman S., Shantsila E. (2014). The Role of Monocytes in Angiogenesis and Atherosclerosis. J. Am. Coll. Cardiol..

[7] Aw N.H., Canetti E., Suzuki K., Goh J. (2018). Monocyte Subsets in Atherosclerosis and Modification with Exercise in Humans. Antioxidants.

[8] Moroni F., Ammirati E., Norata G.D., Magnoni M., Camici P.G. (2019). The Role of Monocytes and Macrophages in Human Atherosclerosis, Plaque Neoangiogenesis, and Atherothrombosis. Mediat. Inflamm..

[9] Hristov M., Weber C. (2011). Differential role of monocyte subsets in atherosclerosis. Thromb. Haemost..

[10] Arnold K.A., Blair J.E., Paul J.D., Shah A.P., Nathan S., Alenghat F.J. (2019). Monocyte and macrophage subtypes as paired cell biomarkers for coronary artery disease. Exp. Physiol..

[11] Moore X.-L., Fang L., Sviridov D., Chin-Dusting J., Andrews K.L., Al-Sharea A., Lee M.K.S., Murphy A.J. (2016). Native LDL promotes differentiation of human monocytes to macrophages with an inflammatory phenotype. Thromb. Haemost..

[12] Stöger J.L., Gijbels M.J., van der Velden S., Manca M., van der Loos C.M., Biessen E.A., Daemen M.J., Lutgens E., de Winther M.P. (2012). Distribution of macrophage polarization markers in human atherosclerosis. Atherosclerosis.

[13] Leitinger N., Schulman I.G. (2013). Phenotypic Polarization of Macrophages in Atherosclerosis. Arterioscler. Thromb. Vasc. Biol..

[14] Timmerman K.L., Flynn M.G., Coen P.M., Markofski M.M., Pence B.D. (2008). Exercise training-induced lowering of inflammatory (CD14+CD16+) monocytes: A role in the anti-inflammatory influence of exercise?. J. Leukoc. Biol..

[15] Child M., Leggate M., Gleeson M. (2013). Effects of Two Weeks of High-intensity Interval Training (HIIT) on Monocyte TLR2 and TLR4 Expression in High BMI Sedentary men. Int. J. Exerc. Sci..

[16] Gano L.B., Donato A.J., Pierce G.L., Pasha H.M., Magerko K.A., Roeca C., Seals D.R. (2011). Increased proinflammatory and oxidant gene expression in circulating mononuclear cells in older adults: Amelioration by habitual exercise. Physiol. Genom..

[17] Ma S., Suzuki K. (2018). Toll-like Receptor 4: Target of Lipotoxicity and Exercise- Induced Anti-inflammatory Effect?. Ann. Nutr. Food Sci..

[18] Stewart L.K., Flynn M.G., Campbell W.W., Craig B.A., Robinson J.P., McFarlin B.K., Timmerman K.L., Coen P.M., Felker J., Talbert E. (2005). Influence of exercise training and age on CD14+ cell-surface expression of toll-like receptor 2 and 4. Brain Behav. Immun..

[19] Mury P., Mura M., Della-Schiava N., Chanon S., Vieille-Marchiset A., Nicaise V., Chirico E.N., Collet-Benzaquen D., Lermusiaux P., Connes P. (2019). Association between physical activity and sedentary behaviour on carotid atherosclerotic plaques: An epidemiological and histological study in 90 asymptomatic patients. Br. J. Sports Med..

[20] Folstein M.F., Folstein S.E., McHugh P.R. (1975). “Mini-Mental State”. A Practical Method for Grading the Cognitive State of Patients for the Clinician. J. Psychiatr. Res..

[21] Crum R.M., Anthony J.C., Bassett S.S., Folstein M.F. (1993). Population-Based Norms for the Mini-Mental State Examination by Age and Educational Level. JAMA.

[22] Gusto G., Vol S., Bedouet M., Leglu C., Decou P., Beslin E., Guillaud C., Copin N., Lantieri O., Tichet J. (2013). Good reproducibility and validity of a self-administered questionnaire evaluating adherence to the French national nutrition and health program recommendations. Press Med..

[23] England C.Y., Andrews R.C., Jago R., Thompson J.L. (2015). A systematic review of brief dietary questionnaires suitable for clinical use in the prevention and management of obesity, cardiovascular disease and type 2 diabetes. Eur. J. Clin. Nutr..

[24] Rosenberg D.E., Norman G., Wagner N., Patrick K., Calfas K.J., Sallis J.F. (2010). Reliability and Validity of the Sedentary Behavior Questionnaire (SBQ) for Adults. J. Phys. Act. Health.

[25] Armstrong T., Bull F. (2006). Development of the World Health Organization Global Physical Activity Questionnaire (GPAQ). J. Public Health.

[26] Bull F.C., Maslin T.S., Armstrong T. (2009). Global Physical Activity Questionnaire (GPAQ): Nine Country Reliability and Validity Study. J. Phys. Act. Health.

[27] Beauchamp C., Fridovich I. (1971). Superoxide dismutase: Improved assays and an assay applicable to acrylamide gels. Anal. Biochem..

[28] Johansson L.H., Borg L.A.H. (1988). A spectrophotometric method for determination of catalase activity in small tissue samples. Anal. Biochem..

[29] Paglia D.E., Valentine W.N. (1967). Studies on the quantitative and qualitative characterization of erythrocyte glutathione peroxidase. J. Lab. Clin. Med..

[30] Pialoux V., Mounier R., Ponsot E., Rock E., Mazur A., Dufour S.P., Richard R.L., Richalet J.-P., Coudert J.D., Fellmann N. (2006). Effects of exercise and training in hypoxia on antioxidant/pro-oxidant balance. Eur. J. Clin. Nutr..

[31] Witko-Sarsat V., Friedlander M., Capeillère-Blandin C., Nguyen-Khoa T., Nguyen A.T., Zingraff J., Jungers P., Descamps-Latscha B. (1996). Advanced oxidation protein products as a novel marker of oxidative stress in uremia. Kidney Int..

[32] Poggio R., Gutierrez L., Irazola V., Rubinstein A.L., Danaei G. (2017). Preventable Ischaemic Heart Disease and Stroke Deaths Attributable to Insufficient Physical Activity: A Comparative Risk Assessment Analysis in the Argentinian Population. Int. J. Sports Exerc. Med..

[33] Arem H., Moore S.C., Patel A., Hartge P., De Gonzalez A.B., Visvanathan K., Campbell P.T., Freedman M., Weiderpass E., Adami H.O. (2015). Leisure Time Physical Activity and Mortality: A detailed pooled analysis of the dose-response relationship. JAMA Intern. Med..

[34] Raftery A.E., Lalic N., Gerland P., Li N., Heilig G. (2014). Joint probabilistic projection of female and male life expectancy. Demogr. Res..

[35] Song P., Fang Z., Wang H., Cai Y., Rahimi K., Zhu Y., Fowkes F.G.R., Fowkes F.J.I., Rudan I. (2020). Global and regional prevalence, burden, and risk factors for carotid atherosclerosis: A systematic review, meta-analysis, and modelling study. Lancet Glob. Health.

[36] Naylor A.R. (2011). Time to rethink management strategies in asymptomatic carotid artery disease. Nat. Rev. Cardiol..

[37] Hansson G.K., Libby P. (2006). The immune response in atherosclerosis: A double-edged sword. Nat. Rev. Immunol..

[38] Kim K.-W., Ivanov S., Williams J.W. (2020). Monocyte Recruitment, Specification, and Function in Atherosclerosis. Cells.

[39] Singh U., Jialal I. (2006). Oxidative stress and atherosclerosis. Pathophysiology.

[40] Seals D.R., Jablonski K.L., Donato A.J. (2011). Aging and vascular endothelial function in humans. Clin. Sci..

[41] Weber C., Shantsila E., Hristov M., Caligiuri G., Guzik T., Heine G.H., Lip G.Y. (2016). Role and analysis of monocyte subsets in cardiovascular disease joint consensus document of the european society of cardiology (Esc) working groups “atherosclerosis & vascular biology” and “thrombosis”. Thromb. Haemost..

[42] Petersen A.M.W., Pedersen B.K. (2005). The anti-inflammatory effect of exercise. J. Appl. Physiol..

[43] Powers S.K., Radak Z., Ji L.L. (2016). Exercise-induced oxidative stress: Past, present and future. J. Physiol..

[44] Smith J.K., Dykes R., Douglas J.E., Krishnaswamy G., Berk S. (1999). Long-term Exercise and Atherogenic Activity of Blood Mononuclear Cells in Persons at Risk of Developing Ischemic Heart Disease. JAMA.

[45] Martinet W., Schrijvers D., De Meyer G. (2011). Necrotic cell death in atherosclerosis. Basic Res. Cardiol..

[46] Pialoux V., Brown A.D., Leigh R., Friedenreich C.M., Poulin M.J. (2009). Effect of Cardiorespiratory Fitness on Vascular Regulation and Oxidative Stress in Postmenopausal Women. Hypertension.

[47] Wewege M.A., Ahn D., Yu J., Liou K., Keech A. (2018). High-Intensity Interval Training for Patients With Cardiovascular Disease—Is It Safe? A Systematic Review. J. Am. Heart Assoc..

[48] Bzowska M., Nogieć A., Skrzeczyńska-Moncznik J., Mickowska B., Guzik K., Pryjma J. (2012). Oxidized LDLs Inhibit TLR-induced IL-10 Production by Monocytes: A New Aspect of Pathogen-Accelerated Atherosclerosis. Inflammation.

[49] Cathcart M.K. (2004). Regulation of Superoxide Anion Production by NADPH Oxidase in Monocytes/Macrophages. Arterioscler. Thromb. Vasc. Biol..

[50] Sohrabi Y., Lagache S.M.M., Schnack L., Godfrey R., Kahles F., Bruemmer D., Waltenberger J., Findeisen H.M. (2019). mTOR-Dependent Oxidative Stress Regulates oxLDL-Induced Trained Innate Immunity in Human Monocytes. Front. Immunol..

[51] Kasapis C., Thompson P.D. (2005). The Effects of Physical Activity on Serum C-Reactive Protein and Inflammatory Markers: A Systematic Review. J. Am. Coll. Cardiol..

[52] Tuan T.-C., Hsu T.-G., Fong M.-C., Hsu C.-F., Tsai K.K.C., Lee C.-Y., Kong C.-W. (2008). Deleterious effects of short-term, high-intensity exercise on immune function: Evidence from leucocyte mitochondrial alterations and apoptosis. Br. J. Sports Med..

[53] Libby P. (2002). Inflammation in atherosclerosis. Nature.

[54] Libby P., Ridker P.M., Maseri A. (2002). Inflammation and Atherosclerosis. Circulation.

[55] Tedgui A., Mallat Z. (2006). Cytokines in Atherosclerosis: Pathogenic and Regulatory Pathways. Physiol. Rev..

[56] Moldoveanu A.I., Shephard R.J., Shek P.N. (2001). The Cytokine Response to Physical Activity and Training. Sports Med..

